# Data mining of immune-related prognostic genes in metastatic melanoma microenvironment

**DOI:** 10.1042/BSR20201704

**Published:** 2020-11-23

**Authors:** Wei Han, Biao Huang, Xiao-Yu Zhao, Guo-Liang Shen

**Affiliations:** 1 Department of Burn and Plastic Surgery, The First Affiliated Hospital of Soochow University, Suzhou, P.R. China, 215000; 2 Department of Surgery, Soochow University, Suzhou, P.R. China, 215000

**Keywords:** biomarkers, immune, melanoma, prognosis

## Abstract

Skin cutaneous melanoma (SKCM) is one of the most deadly malignancies. Although immunotherapies showed the potential to improve the prognosis for metastatic melanoma patients, only a small group of patients can benefit from it. Therefore, it is urgent to investigate the tumor microenvironment in melanoma as well as to identify efficient biomarkers in the diagnosis and treatments of SKCM patients. A comprehensive analysis was performed based on metastatic melanoma samples from the Cancer Genome Atlas (TCGA) database and ESTIMATE algorithm, including gene expression, immune and stromal scores, prognostic immune‐related genes, infiltrating immune cells analysis and immune subtype identification. Then, the differentially expressed genes (DEGs) were obtained based on the immune and stromal scores, and a list of prognostic immune‐related genes was identified. Functional analysis and the protein–protein interaction network revealed that these genes enriched in multiple immune-related biological processes. Furthermore, prognostic genes were verified in the Gene Expression Omnibus (GEO) databases and used to predict immune infiltrating cells component. Our study revealed seven immune subtypes with different risk values and identified T cells as the most abundant cells in the immune microenvironment and closely associated with prognostic outcomes. In conclusion, the present study thoroughly analyzed the tumor microenvironment and identified prognostic immune‐related biomarkers for metastatic melanoma.

## Introduction

Skin cutaneous melanoma (SKCM) is a common skin malignancy with high metastasis and mortality rates [1]. Once melanoma has spread, this type of cancer rapidly becomes life-threatening. Although significant progress in the treatments of advanced melanoma has achieved some level of success during the past several years, patients with metastatic melanoma still show poor prognosis [2]. Immunotherapy was regarded as a foundation in melanoma treatment, and is assumed to regulate host immunity against the tumor [3,4]. Immunotherapeutic strategies, including anti-PD1 and anti-CTLA4, have achieved some success and improved patients’ survival; however, still many patients don’t show a long-lasting response and tend to have poor prognosis [5].

Currently, tumor microenvironment (TME) studies have raised hopes for improved treatment of melanoma patients, especially for patients with metastatic lesions [6]. There is evidence revealing that increased density of tumor-infiltrating lymphocytes (TILs) are correlated with better outcomes, as well as a reduced occurrence of lymph node metastasis and a lengthier disease-free survival (DFS) [7]. Thomas et al. found that the degree of lymphocyte infiltration was an independent prognosticator of DFS, indicating that a lesser grade was related to a decreased DFS [8]. These researches implied that assessing the diversity of the TME and transforming the immune microenvironment can be a promising strategy for melanoma therapy. Due to the complexity and heterogeneity of the genomic landscape among different types of tumors, it is urgent to investigate the TME in melanoma as well as to identify efficient biomarkers in the diagnosis and treatments of SKCM patients. ESTIMATE algorithms have been developed to predict tumor purity in various cancers based on the specific gene expression signature of immune and stromal cells [9]. In the present work, we explored the TCGA database and ESTIMATE algorithm to identify immune‐related prognostic biomarkers for metastatic melanoma.

## Materials and methods

### Gene expression datasets

Gene expression profile for metastatic SKCM patients (*n*=368) was obtained from the TCGA data portal (https://tcga-data.nci.nih.gov/tcga/). Clinical data (*n*=371), including gender, age, pathological stages, Breslow depth, Clark level and survival status, were also downloaded from the TCGA data portal [10]. Immune scores and stromal scores were calculated by applying the ESTIMATE algorithm to the downloaded database [9]. For validation, GSE59334 (*n*=74) containing expression profiling of melanoma patients with clinical information were obtained from the Gene Expression Omnibus (GEO) database (https://www.ncbi.nlm.nih.gov/geo/) [11]. The clinicopathological characteristics of the metastatic SKCM patients from the TCGA database was shown in Table 1.

**Table 1:** Clinicopathological characteristics of metastatic SKCM patients

Characteristics	TCGA cohort (*N*=371)
*N* (%)	
Age	
y ≤ 60 years	216 (59.7)
>60 years	146 (40.3)
Gender	
Male	232 (62.7)
Female	138 (37.3)
Clark level	
I	6 (2.4)
II	17 (6.8)
III–IV	193 (77.5)
V	33 (13.3)
Breslow depth (mm)	
≤0.75	36 (9.8)
0.76–1.50	170 (46.1)
1.51–4.00	96 (26.0)
>4.00	67 (18.1)
pT stage	
T1-T2	115 (43.4)
T3-T4	150 (56.6)
pN stage	
N0	178 (62.7)
N1	67 (23.6)
N2	39 (13.7)
pM stage	
M0	322 (93.6)
M1	22 (6.4)
Pathologic stage	
I-II	150 (47.5)
III-IV	166 (52.5)

### Identification of differentially expressed genes

All samples were divided into high/low immune‐score as well as stromal‐score groups based on the ESTIMATE results. Data analysis was performed using the limma package in R software. |log(Fold change)| > 1.5, *P*<0.05 and FDR<0.05 were set as the cutoffs to screen for DEGs. Heatmaps were generated using the pheatmap package.

### Survival curve

The Kaplan–Meier methods were used to illustrate survival differences between the low- and high-stromal/immune score groups with overall survival (OS) and recurrence‐free survival (RFS) and identify the prognostic genes. The *P*-value < 0.05 was set as the cut-off value.

### Function enrichment analysis

Gene Ontology (GO) analysis and the Kyoto Encyclopedia of Genes and Genomes (KEGG) analysis of DEGs and prognostic genes were performed by DAVID (The Database for Annotation, Visualization and Integrated Discovery) [12]. False discovery rate (FDR) < 0.05 and *P*<0.01 were used as the cut-offs.

### PPI network construction and model analysis

The protein–protein interaction (PPI) network was built using STRING and visualized in Cytoscape software [13]. The plug-in MCODE was then used to find clusters based on the topology to locate densely connected regions. Individual modules with at least 15 nodes and 100 edges were selected for further analysis.

### Estimation of immune cell type fractions

To quantify the proportions of immune cells, 238 prognostic gene expression data were used to measure the abundance of several types of infiltrating immune cells (B cells, T cells, NK cells, monocytes, macrophages, myeloid dendritic cells, mast cells, eosinophils and neutrophils) in melanoma patients using CIBERSORT algorithm [14]. And the number of samples used for clustering analyses was 368 from the TCGA database. Then, we carried out unsupervised clustering of the infiltrating immune cells, dividing patients into different immune subtypes based on the *k*‐means. Combining immune-score and survival analysis, high‐risk and low-risk immune subtypes were identified. Statistical analysis and graphical plotting were conducted using R software.

### Statistical analysis

One‐way analysis of variance was applied to compare the immune and stromal scores in different groups by using GraphPad Prism 8. Differential analysis of expressed genes, functional analysis as well as unsupervised clustering analysis were all performed in R software.

## Results

### Association of immune scores and stromal scores with SKCM prognosis

**Figure 1:**
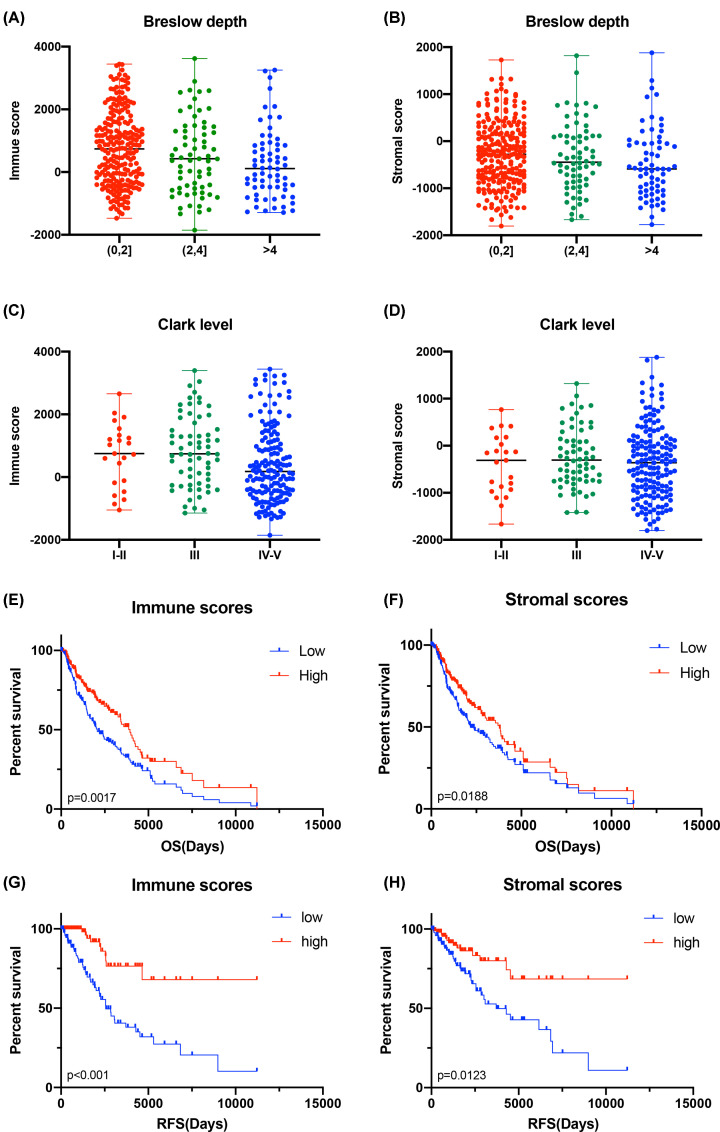
Immune scores and stromal scores are associated with metastatic melanoma prognosis (**A** and **B**) The distribution of immune and stromal scores were significantly associated with Breslow depth (*P*=0.0034, *P*=0.00453). (**C** and **D**) The distribution of stromal scores did not vary across different Clark levels (*P*=0.5299), while the distribution of immune scores did differ (*P*=0.0124). (**E**) The analysis of patients’ overall survival (OS) based on immune scores. (**F**) The analysis of patients' OS based on stromal scores. (**G**) The analysis of patients' recurrence‐free survival (RFS) based on immune scores. (**H**) The analysis of patients’ RFS based on stromal scores.

In general, the immune score and stromal score were significant associated with metastatic melanoma prognosis. Based on the ESTIMATE algorithm, immune scores ranged from -1853.02 to 3622.19, and stromal scores were distributed between -1804.83 and 1879.32. The distribution of immune and stromal scores was significantly associated with Breslow depth (*P*=0.0034, *P*=0.00453) (Figure 1A,B). As shown in Figure 1C,D, the distribution of stromal scores did not vary across different Clark levels (*P*=0.5299), while the distribution of immune scores did differ (*P*=0.0124).

To investigate the correlation of OS and RFS with immune scores and stromal scores, we divided the 368 metastatic SKCM cases into high or low-score groups based on their immune and stromal scores. Kaplan–Meier survival curves indicated that immune scores were positively related to both OS (*P*=0.0017) and RFS (*P*<0.001). Similarly, we found that stromal scores were also positively associated with both OS (*P*=0.0188) and RFS (*P*=0.0123), suggesting that both are positive factors for the prognosis of patients with metastatic melanoma (Figure 1E–H).

### Comparison of gene expression profile and function annotation

**Figure 2:**
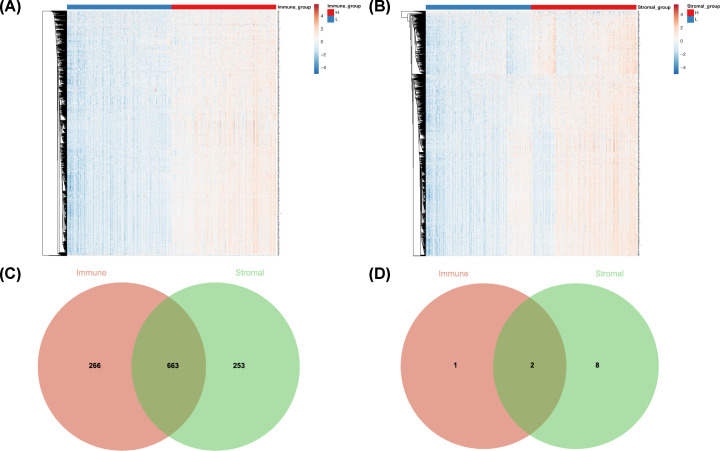
Comparison of gene expression profile with immune and stromal scores of melanoma (**A** and **B**) Heatmaps of gene expression profiles of samples between high and low immune scores/stromal scores groups. (**C** and **D**) Venn diagram analysis of aberrantly expressed genes based on immune and stromal scores.

Heatmaps in Figure 2A,B showed different gene expression profiles of samples between high and low immune/stromal scores groups. For comparison based on immune scores, 929 genes were up-regulated and 3 genes down-regulated in the high score (|logFC| >1.5; *P*<0.05, Figure 2C). Similarly, 916 up-regulated genes and 10 down-regulated genes were selected according to the stromal scores (|logFC| >1.5; *P*<0.05, Figure 2D).

**Figure 3:**
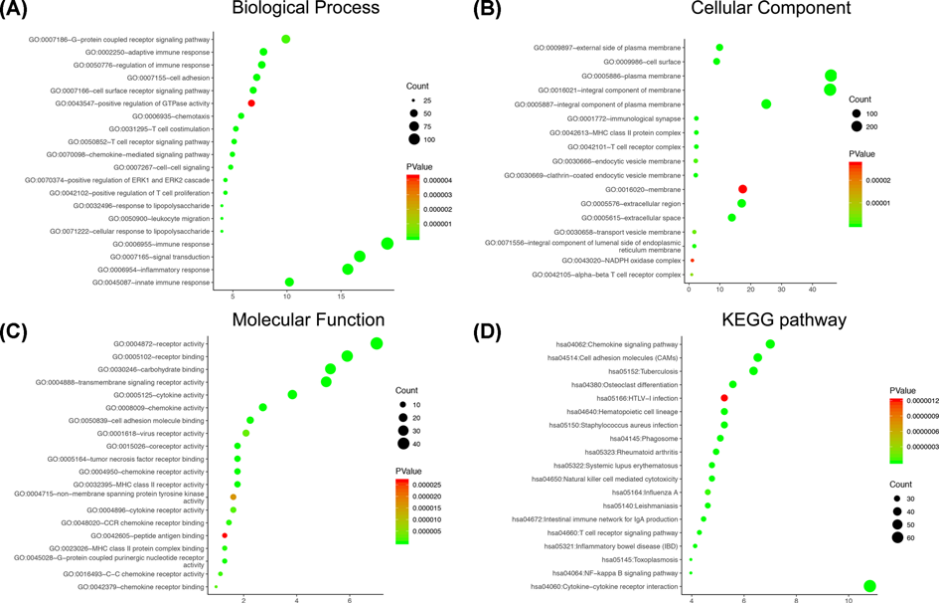
The functional analysis of immune‐related genes (**A–C**) GO analysis (BP, CC, MF) of immune‐related genes. (**D**) KEGG analysis of immune‐related genes; GO, Gene Ontology; BP, biological process; MF, molecular function; CC, cellular component; KEGG, Kyoto Encyclopedia of Genes and Genomes.

Next, we selected the 663 overlap up-regulated genes for further study. Functional enrichment clustering of overlap genes showed a strong association with immune response. GO analysis indicated that changes in biologic processes significantly enriched in immune response, inflammatory response, signal transduction, innate immune response and adaptive immune response (Figure 3A). Changes in cellular components were mainly enriched in the plasma membrane, integral component of plasma membrane, external side of plasma membrane, integral component of membrane and T-cell receptor complex (Figure 3B). As for the molecular function, changes were mostly enriched in receptor activity, carbohydrate binding, chemokine activity, MHC class II receptor activity and transmembrane signaling receptor activity (Figure 3C). In addition, all the pathways yielded from the KEGG analysis (Figure 3D) were closely associated with immune response, such as cytokine–cytokine receptor interaction, chemokine signaling pathway and natural killer cell mediated cytotoxicity.

### Overall survival of overlapped genes

**Figure 4:**
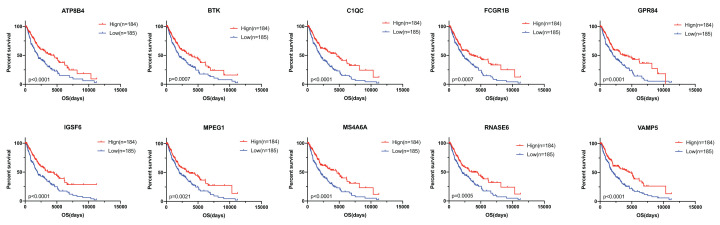
Kaplan–Meier survival curves for immune‐related genes associated with overall survival

Subsequently, we performed survival analysis to examine the overlapped 663 up-regulated genes in order to identify prognostic genes, and 600 genes (90.5%) were correlated with better overall survival (*P*<0.05, Figure 4), which were considered potential prognostic immune‐related genes for further study.

### Validation in the GEO database

**Figure 5:**
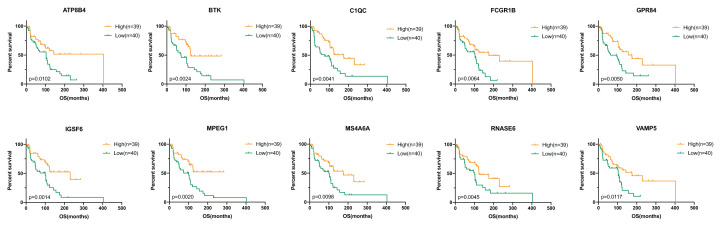
Validation of the TCGA results in other cohorts from the GEO database Kaplan–Meier survival curves for the verified genes associated with overall survival.

To find out whether the genes identified from the TCGA database also are of prognostic significance in additional GEO cases, we downloaded and analyzed gene expression data of GSE59334 from GEO database. A total of 239 genes in TCGA cohort were validated (Figure 5 and Supplementary Table S1) to be significantly linked to a better prognosis, which might act as potential prognostic biomarkers for melanoma patients.

### Protein–protein interactions among prognostic genes

**Figure 6:**
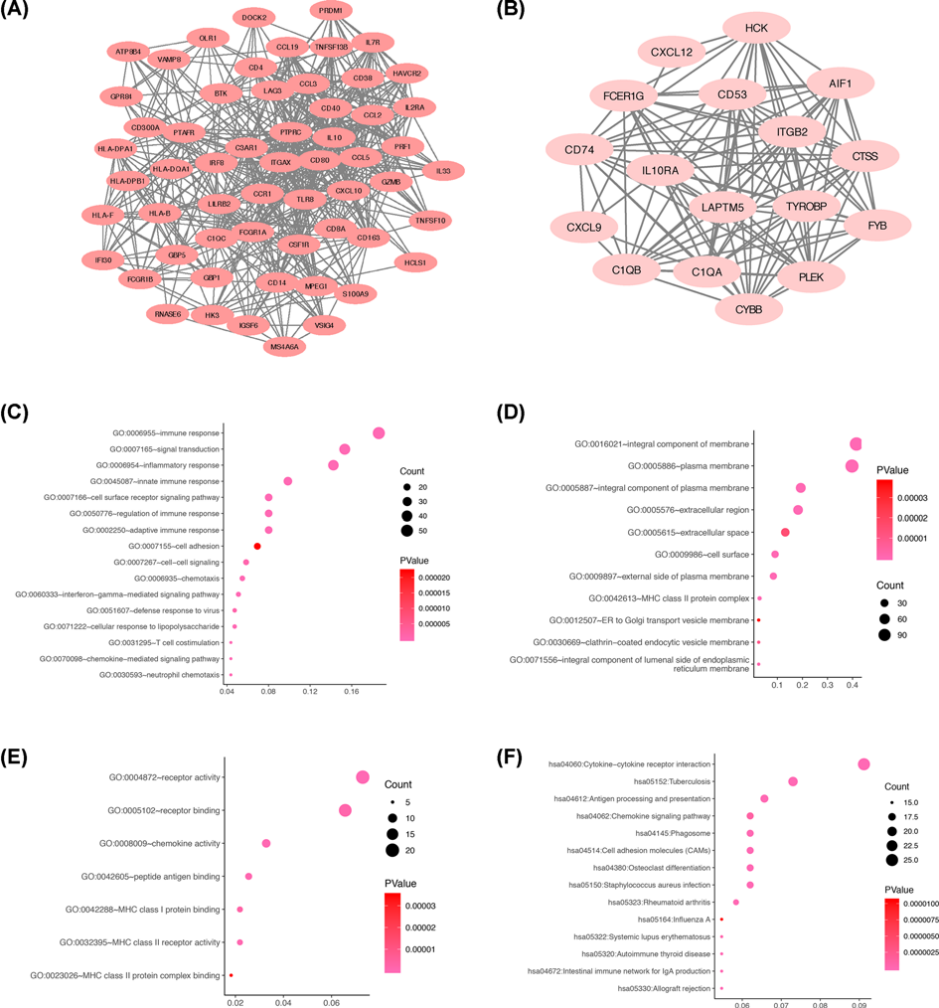
The functional analysis of prognostic immune‐related genes (**A** and **B**) Top two modules in the PPI networks. (**C–E**) GO analysis of prognostic immune‐related genes. (**F**) KEGG analysis of prognostic immune‐related genes; GO, Gene Ontology; KEGG, Kyoto Encyclopedia of Genes and Genomes.

To better understand the interplay among the identified prognostic genes, we obtained protein–protein interaction (PPI) networks among 239 them, including 211 nodes and 2499 edges. We selected two modules with at least 15 nodes and 100 edges for further analysis. In module A (Figure 6A), C1QC, MPEG1, IL7R, CD300A, HAVCR2, CD38, TLR8, and CXCR4 had higher connectivity degree values, indicating they were the core genes in the module. In module B (Figure 6B), PLEK, LAPTM5, C1QA, HCK, CYBB, and ITGB2 were remarkable for having many connections with other genes. Moreover, BP analysis indicated that these prognostic genes were mainly involved in immune response and inflammatory response. CC showed that changes were primarily enriched in plasma membrane, external side of plasma membrane and integral component of plasma membrane. As for MF, changes were mostly in receptor activity, chemokine activity and MHC class II receptor activity. KEGG analysis showed that these genes participated in cytokine–cytokine receptor interaction, antigen processing and presentation and cell adhesion molecules (CAMs) (Figure 6C–F).

### Immune components estimation and subtype analysis

**Figure 7:**
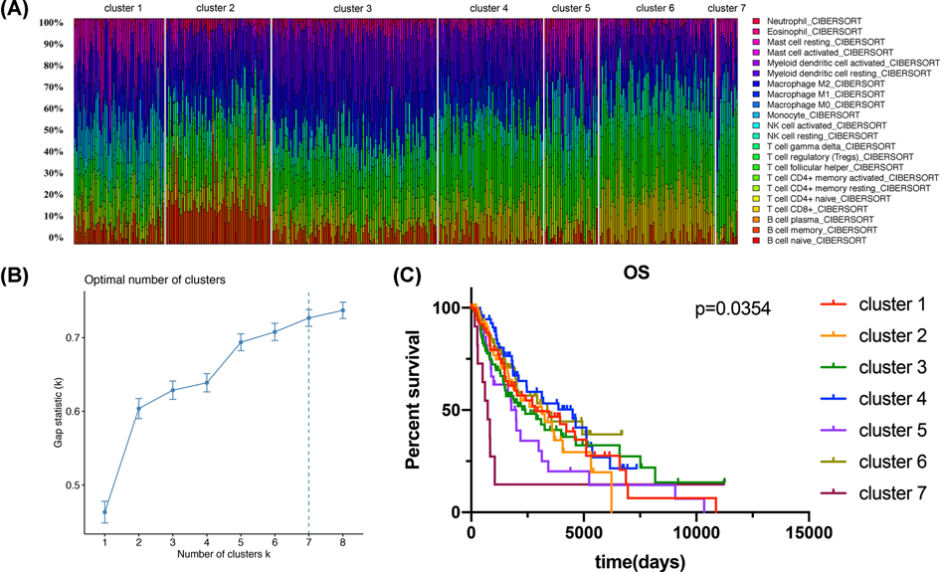
The immune landscape of metastatic melanoma microenvironment (**A**) Unsupervised clustering of all samples based on immune‐cell proportions. Stacked bar charts of samples ordered by cluster assignment. (**B**) Optimal numbers of clusters. (**C**) Survival analysis of patients within different subtypes.

The expression data of prognostic genes were used to predict immune cell profiling in metastatic melanoma. The cell proportions of each cluster are shown in Figure 7A. We undertook unsupervised clustering with the *k*‐means algorithm of all samples based on the immune‐cell proportions. The optimal number of clusters was 7 (Figure 7B), thus seven immune subtypes were identified. T cells were the most abundant cells in the immune microenvironment. Moreover, clusters were associated with distinct patterns of survival (*P*=0.0354, Figure 7C), indicating that though T cells were the most abundant cells in the microenvironment, other immune cells had a stronger influence in patients’ prognosis. Cluster 4, defined by high levels of T cells and a few macrophages, and cluster 6, defined by a high level of T cells with a few B cells, were both associated with better prognosis. In contrast, cluster 5 and cluster 7 (both defined by high levels of T cells, mast cells, and neutrophils) were associated with a poor prognosis than other clusters.

## Discussion

SKCM is one of the deadly human malignancies, and its metastasis and mortality is high [1]. Nowadays, there is still a lack of effective prognostic biomarkers that could serve to guide cancer therapy. It is widely acknowledged that the immunological features of the TME play important role in the evaluation of tumor prognosis [15]. Therefore, the present study aims to identify immune‐related prognostic genes that could better patients’ overall survival by exploring the TME.

ESTIMATE algorithm was developed to calculate the tumor purity by immune and stromal scores. It was soon applied in glioblastoma, breast cancer and colon cancer, showing the effectiveness of such big-data based algorithms [16–18]. In our study, both of immune, stromal scores were found differently distribute in Breslow depth and Clark level, which were important prognostic indicators for SKCM patients. In addition, high levels of immune/stromal scores were related to better OS as well as RFS. Next, all of the samples were divided into high and low immune/stromal groups and 663 immune-related genes were identified. Top GO terms in the biological process included immune response, inflammatory response, signal transduction, innate immune response and adaptive immune response In addition, all the pathways that were yielded from KEGG analysis were associated with immune response. Consistent with previous studies, our findings verified that immune cells and extracellular matrix play significant roles in building the melanoma microenvironment [19–21]. Furthermore, we performed the overall survival analysis of 663 immune-related genes and identified that 600 genes (90.5%) were associated with better outcomes in metastatic melanoma patients. PPI network and two significant models were constructed among them and highly connected nodes in the models, including IL7R, CD38 and CXCR4 were closely associated with immune response, especially participating in regulating CD4+ and CD8+ T cells [22–24].

For validation, GEO dataset was download and 239 immune-related prognostic genes were identified with better survival outcomes. We plotted the survival curves of 10 genes that have not been studied previously in melanoma. Among them, we particularly interested in C1QC and MPEG1, the most highly interconnected genes in the PPI network. A previous study showed that C1q plays a fundamental role in the pathogenesis of gliomas and closely associated with the prognosis in a diverse grade of gliomas [25]. C1QC encodes the C‐chain polypeptide of Complement C1q, widely expressed in the TME among various types of human malignancies [26], suggesting that C1QC could also be a significant factor in melanoma. MPEG1 (macrophage-expressed gene 1) is observed to be overexpressed in multiple human cancer tissues, including breast, liver, pancreas, lung and thyroid [27]. The depletion of MPEG1 could impact cell mitosis, disturb centrosome duplication, as well as induce chromosome misalignment and mis-segregation in hepatocellular carcinoma [28,29]. ATP8B4, a participant in ATP biosynthesis and phospholipid transport via a variety of potential mechanisms, was found to be a risk factor for systemic sclerosis and pulmonary vascular complications [30]. BTK, located in the downstream of signal transduction of B-cell antigen receptor, might be a useful biomarker to predict the prognosis of lung cancer patients [31]. FCGR1B was overexpressed in renal cell carcinoma, predicting poor patient prognosis [32]. GPR84, a protein-coding gene of the metabolic G protein-coupled receptor family, can be regarded as a potential signature for the prognostic prediction of hepatocellular carcinoma [33]. IGSF6 was considered to be closely related to the susceptibility of inflammatory bowel disease [34]. In ovarian cancer, MS4A6A was associated with pathological grade and might act as a surface marker for M2 macrophages [35]. The down-regulated RNASE6 might be a prognostic biomarker for esophageal squamous cell carcinoma [36]. VAMP5 was observed to be correlated in tumor microenvironment of brain lower grade glioma [37]. Taken together, all the 10 selected prognostic genes, shown in Figure 5, are confirmed to be closely associated with immune diseases and human malignancies, suggesting it might act as an important role in melanoma as well.

Finally, we used expression data of the prognostic genes to assess immune cell profiling in metastatic melanoma by CIBERSORT algorithm. A previous study suggested that the imbalance of immune cell component was strongly associated with worse outcomes [38]. In the present study, seven immune subtypes were identified by unsupervised clustering based on the abundance of immune cells, and the subtypes underwent survival analysis. Among them, we found that T cells were the most abundant. CD8+ T cells mainly recognize MHC-I antigens and produce granzymes and perforin to kill tumor cells [39]. Taggart et al. found the important role of CD8+T cells in enhancing the efficacy of anti-PD-1/anti-CTLA4 in melanoma brain metastases [40]. Increasing evidence revealed that an elevated proportion of CD8+ T cells may improve the melanoma prognosis as well as reducing the risk values [41]. However, the role of CD4+T cells (MHC class II) in the TME is always been neglected and the immune response of single CD8+T cells is not enough to eliminate tumor cells In actual immunotherapy [42]. Recent studies have found that CD4+T cell responses are required for optimal priming of MHC-I restricted CD8+T cells and their maturation into cytotoxic T lymphocytes [43]. Moreover, CD4+T cells play an important role in the TME by secreting IFN-γ, increasing CD8+T cells number and enhancing their lethality [44,45]. Therefore, we predicted CD4+T cells, CD8+T cells and exploration of their proportion will be of great value to improve objective response rate for metastatic melanoma treatment. However, further experiments, as well as validated cohorts, were required to clarify the absoluteness of these findings.

## Conclusion

Our study explored the TME based on TCGA and GEO cohorts in order to better understand potential immune effects and the mechanism in metastatic melanoma. A comprehensive analysis was performed including immune and stromal scores, gene expression, prognostic immune‐related genes, infiltrating immune cells analysis and immune subtype identification. Our results provide insight into metastatic melanoma microenvironment and identified the immune-related prognostic genes. Importantly, the evaluation of immune subtypes and infiltrating cells might help us better explore the melanoma mechanism and immunotherapies. However, further studies are needed to unravel the functional mechanism behind these immune-related prognostic genes in the metastatic melanoma microenvironment.

## Supplementary Material

Supplementary Table S1

## Data Availability

The datasets analyzed for this study can be found in the GEO (http://www.ncbi.nlm.nih.gov/geo) and TCGA (https://www.cancer.gov).

## Abbreviations

CAM: cell adhesion molecule
DEG: differentially expressed gene
GEO: Gene Expression Omnibus
OS: overall survival
PPI: protein–protein interaction
RFS: recurrence‐free survival
SKCM: skin cutaneous melanoma
TCGA: the Cancer Genome Atlas
TIL: tumor-infiltrating lymphocyte
TME: tumor microenvironment
