# Supplementary material for: Data mining of immune-related prognostic genes in metastatic melanoma microenvironment
Source: Biosci Rep. 2020 Nov 23;40(11):BSR20201704. doi: 10.1042/BSR20201704 (PMC7685010; doi:10.1042/BSR20201704)
Supplement: Supplementary Table S1 [file BSR-2020-1704_supp.pdf]

Supplementary table 1. Prognostic genes verified by GEO dataset.

**Prognostic Genes**	**HR**
SLCO2B1	0.60505
HSD11B1	0.384452
LRRC61	4.007592
MS4A6A	0.65831
STX11	0.544649
ANKRD22	0.475556
IGSF6	0.516244
FAM49A	0.086638
LILRB3	0.643085
ARHGAP25	0.552454
ITGB2	0.70331
SLC7A7	0.669714
SLC15A3	0.701642
ITM2A	1.765239
SIGLEC10	0.644789
CLEC4A	0.399898
SLAMF8	0.603274
KCNK6	0.391132
APOL3	0.687694
TNFAIP2	0.464411
SUCNR1	0.16982
ADAMDEC1	0.744828
CECR1	0.753776
APOL1	0.141763
IL18RAP	0.368662
COL4A4	0.109564
AMICA1	0.590893
FCGR1B	0.636735
GPR84	0.381098
PLCG2	0.682018
LST1	0.643877
SERPINA1	0.706754
SQRDL	0.536502
SLC2A5	0.675308
ATP8B4	0.598324
NCF1C	0.641524
ASGR2	0.170004
SLC27A2	0.093858
VSIG4	0.682413

PLXDC2	0.580565
VNN1	0.068555
MYO1F	0.476751
FGD2	0.739665
SAMSN1	0.628867
OCIAD2	1.564141
TNFRSF17	0.635461
FAM113B	0.720796
FCRL5	0.413591
AOAH	0.54087
GVIN1	0.742176
FCER1G	0.788608
LILRA5	0.232639
BCL2L14	1012.284
EAF2	0.523803
GIMAP6	0.661575
HCLS1	0.775611
JSRP1	0.725437
LAX1	0.624205
IL18R1	0.436304
RNASE6	0.649113
GAB3	0.222568
DENND3	0.448001
MPEG1	0.30073
HTRA4	0.535988
CD300A	0.655903
ZBED2	0.305945
CYSLTR1	0.392954
TNIP3	0.102326
DNAJC5B	0.024952
P2RY6	0.471682
VAMP5	0.799604
HLA-DQA1	0.721227
LAPTM5	0.629412
IFI30	0.613705
HLA-DRA	0.705242
KLRC1	0.010024
RASSF4	0.490238
HLA-DPA1	0.715017
IL10	0.000522
NCF4	0.498216
KIR3DL2	0.012582

CD74	0.724192
C1QC	0.695409
KIR2DL4	0.374233
RARRES1	0.53155
PLEK	0.690032
TLR8	0.483666
HLA-DMA	0.68321
HLA-DMB	0.713172
IL2RA	0.300248
LYN	0.66874
CD14	0.702826
CCL8	0.723006
CCR1	0.37144
CD84	0.441255
FCGR1A	0.466944
C1QB	0.735972
PIM2	0.697148
C1QA	0.680616
CD40	0.452132
OLR1	0.548666
SYK	0.625426
CR1	0.028048
CD163	0.68906
IL15RA	0.134257
CD38	0.6251
CD80	0.149595
MS4A7	0.531073
SPINT2	0.567502
CYBB	0.642709
GCH1	0.393307
IL18BP	0.72035
LYZ	0.784769
CD72	0.509468
AIF1	0.680121
RASSF5	0.646533
CASP5	0.030439
LCP1	0.775657
UCP2	0.672403
PTAFR	0.31565
CCL5	0.806543
LILRB4	0.620396
HCK	0.593704

IL33	0.597592
IRF1	0.723884
MGC29506	0.761551
MYO7A	0.338017
GNLY	0.626113
CD300LF	0.541445
GZMB	0.746073
ARHGAP30	0.54669
IRF8	0.744576
ALOX5	0.726037
KLHL6	0.420869
HLA-F	0.688872
CXCR6	0.438067
HAVCR2	0.70005
CD86	0.648392
TYROBP	0.75214
HAMP	0.678273
BTK	0.592986
CSF1R	0.716807
CMKLR1	0.36108
CD33	0.528095
HLA-DPB1	0.624388
SOCS1	0.45466
LILRB1	0.095148
LILRB2	0.612872
EFEMP1	0.756356
HLA-B	0.767565
S100A9	0.775002
CCL3L1	0.555289
TNFSF13B	0.722704
PILRA	0.42507
CCL19	0.818209
PTPRE	0.572578
VAMP8	0.736308
CFB	0.657348
PLA2G7	0.71284
OSCAR	0.542599
GRIN3A	0.036732
IL10RA	0.60658
CD53	0.63237
PTPN6	0.683136
DOCK2	0.725767

CTSS	0.587583
VAV1	0.437688
TNFSF10	0.703192
SRGN	0.767523
BIRC3	0.741687
TAGAP	0.293355
P2RY13	0.59395
PTPRC	0.350039
HLA-DRB6	0.72016
NKG7	0.768893
CD226	0.080444
IL2RB	0.702265
WAS	0.740403
HLA-DOA	0.735543
PIK3CG	0.42284
PRF1	0.665178
CXCL12	0.772709
CCL3	0.645147
CAMK1D	0.234047
C3AR1	0.474403
HK3	0.565416
GBP5	0.764078
CASP1	1423.696
IGJ	0.82007
RARRES3	0.80081
BATF	0.630306
CXCL9	0.844117
SLAMF6	0.747359
CD4	0.594452
ABCG1	3.507901
EPSTI1	0.790331
SLAMF1	0.562455
PRDM1	0.152535
IL1R2	0.49745
FBP1	0.720964
FAM46C	0.7941
MAPK13	0.637812
SLA	0.689358
CXCL10	0.847043
IL4I1	0.665564
GZMA	0.748227
AQP9	0.687862

APBB1IP	0.69238
ITGAX	0.67411
KCNN3	0.050119
LOC400759	0.521691
GBP1	0.810407
CASP10	0.020334
SLAMF7	0.362288
IL7R	0.729432
CD8A	0.803031
FMNL1	0.081833
SIGLEC7	0.098299
FYB	0.78785
NAPSB	0.747988
CSTA	0.012683
LAG3	0.755757
PARVG	0.691103
CD52	0.808168
CCL2	0.775848
TDO2	0.581094
GNA15	0.663233
HCST	0.790062
XAF1	0.585467
MSR1	0.371624
TAP1	0.766783
LRMP	0.633214
FPR1	0.696531
FCRL2	0.604739
CD244	0.037307
LAT2	0.627627
SEMA4D	0.750198
S100A8	0.81744
